# Improving Spent Coffee Biochar for Effective Organic Contaminant Removal from Aqueous Media

**DOI:** 10.1021/acsomega.4c09171

**Published:** 2025-01-30

**Authors:** Inga Block, Harshadrai M. Rawel, Tillmann Klamroth, Christina Günter, Jiyong Kim, Fabian Loepthien, Shashank K. Gahlaut, Ilko Bald, Andreas Taubert

**Affiliations:** † Institute of Chemistry, University of Potsdam, Karl-Liebknecht-Straße 24-25, D-14476 Potsdam, Germany; ‡ Institute of Nutritional Science, University of Potsdam, Arthur-Scheunert-Allee 114-116, D-14558 Nuthetal, Germany; § Institute of Earth and Environmental Sciences, University of Potsdam, Karl-Liebknecht-Straße 24-25, D-14476 Potsdam, Germany; ∥ Fraunhofer Institute for Applied Polymer Research (IAP), Geiselbergstrasse 69, D-14476 Potsdam, Germany

## Abstract

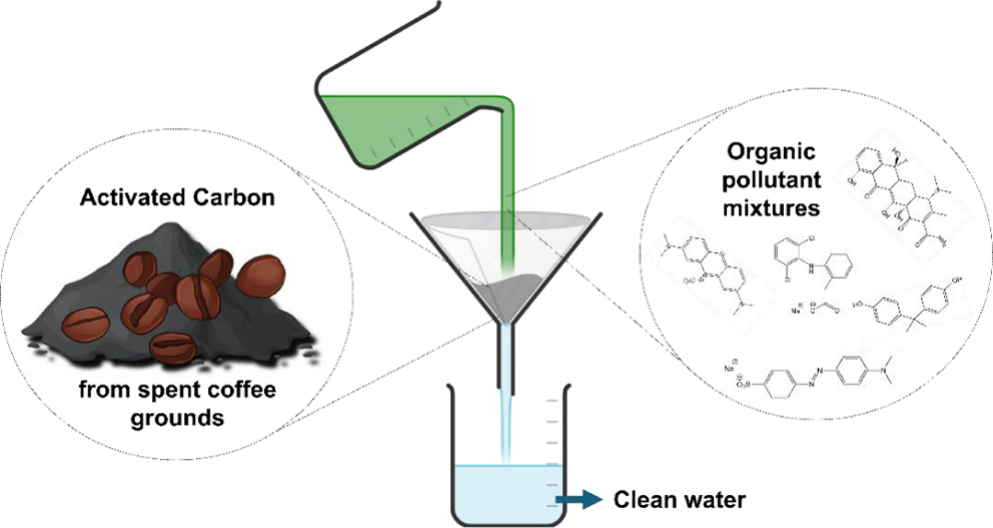

The contamination of (waste)water with organic pollutants, such as pharmaceuticals and dyes, is drastically increasing. Their removal process presents several difficulties, and often activated carbon (AC) is used in a filtration step. While commercial AC is often based on fossil resources, in this study, we present a new approach toward biochar from spent coffee (SC). This new AC has considerably enhanced surface areas and porosities, making it suitable for wastewater treatment. Using MgCO_3_ as an activating agent, a biochar with a significantly enhanced surface area of ∼600 m^2^/g is produced in a simple but efficient manner. The resulting biochar is effective for the removal of a whole spectrum of organic pollutants in aqueous systems. The dyes methylene blue (MB) and methyl orange (MO), but also the pharmaceuticals diclofenac (DCF) and tetracycline (TET), as well as the xenoestrogen bisphenol A (BPA), are successfully removed by up to 100% from aqueous solutions with the new adsorbents. Removal efficiencies depend on the pH of the solutions. In contaminant mixtures, the biochar shows preferences for adsorption toward some compounds but still shows very high adsorption capacities for all contaminants.

## Introduction

Reactive or water-soluble dyes, like methylene blue (MB) or methyl orange (MO), present a hazard in countries or regions with large textile or dyeing industries when discharged directly into surface water streams. In addition, pharmaceuticals such as diclofenac (DCF), antibiotics such as tetracycline (TET), or the plasticizer bisphenol A (BPA), an endocrine disruptor, are contaminants found in small concentrations in surface waters throughout the world. Their quasi-persistent nature and the fact that they are often difficult to remove by wastewater treatment plants generate tremendous environmental and health problems.^1−5^ One approach for removing these micropollutants in wastewater treatment plants is the use of special activated carbons (ACs) in an additional filtration step.^1,6−8^ Here, biochars based on agricultural wastes present an alternative to the currently commercially available ACs made out of coal, petroleum, coconut husks, or wood.^7,9,10^

Plenty of materials made from biological waste have already been studied for the production of biochar with applications in catalysis, electrochemistry, soil remediation, or water decontamination.^11,12^ Biomaterials like pine cones, peach stones, apple waste, straw, sunflower oil cake, or fruit seeds have been thoroughly studied as raw materials.^13−20^ One of the main advantages of agricultural waste is its local availability in many different forms; this generally avoids long transport, which, in turn, avoids the use of fossil resources for biochar AC fabrication.

For this work, we focus on spent coffee grounds (SC) as a biochar resource. In Germany alone, the average coffee-drinking person consumes 3.3 cups per day.^21^ According to the German Coffee Association in 2022, each German citizen consumed 167 L of coffee.^22^ Now, assuming that for each cup of 125 mL of coffee, 8 g of coffee grounds are used, 1336 cups of coffee are consumed, producing 10.7 kg of SC per person per year. Overall, for the Federal Republic of Germany, with a population of 84 million people, this equals a production of ca. 90 kilotons of SC per year, making it an abundant biowaste material. On a global scale, 11.14 million tons of spent coffee grounds were produced in 2022/2023.^23^

Typically, the synthesis of bioderived ACs includes the chemical activation of the raw materials using either KOH, acids, or ZnCl_2_ followed by pyrolysis. Among others, the activation and synthesis methods mentioned above have also been applied to SC and other coffee-related biowastes.^24−34^Table 1 compiles some examples of SC activation processes and contaminants that have been removed from water with the respective materials.

**Table 1:** Activation Methods for SC in Biochar Synthesis and Contamination Removal Were Studied Including the Respective Removal Efficiencies

Pretreatment	*T*_Pyr_ [°C]	*a*_BET_ [m^2^/g]	System	Adsorption capacity	Removal efficiency [%]	Publication
ZnCl_2_	500	1039	BPA	123.20 mg/g	98	(24)
citric acid	-	-	Cu(II), Pb(II)	0.77/1.53 mmol/g (Pb(II)/Cu(II))	-	(25)
H_2_SO_4_	600	146	iron, *ortho*-phosphate	-	77–84	(26)
CaCO_3_	850	167	fermentation residues	13.99 mg/g	≤80	(28)
H_2_O (vapor)	800	464	Ni(II)	51.91 mg/g	≤70	(34)
KOH	700–900	≤1040	methane storage	4.20 mmol/g	-	(30)
formaldehyde	-	-	Cu(II), Cr(VI)	70/45 mg/g (Cu(II)/Cr(VI))	60–75	(31)
H_3_PO_4_	500	1110	ethylene, *n*-butane	84 cm^3^/g	-	(33)

Previously, we introduced a rather simple method to prepare an effective biochar adsorbent based on SC using CaCO_3_ as an activator for pyrolysis.^28,35^ The addition of CaCO_3_ leads to rather porous powders with surface areas below 200 m^2^/g that effectively remove organic dyes from aqueous solutions.^35^

In the current study, a new and significantly improved approach is provided by using MgCO_3_ resulting in a further enhanced SC adsorbent. The resulting new biochar has a surface area about three times as large as before and is much more effective in adsorption processes. As organic pollutants, we focus on the organic dyes MB and MO, with MB especially playing a relevant role in fabric industries and often being discharged into rivers as is, as well as on the adsorption of other common organic pollutants, i.e., DCF, BPA, and TET. Depending on the composition and nature of the water body, its pH value will not necessarily be neutral but can be acidic or basic as well. The study of the adsorption behavior of each single contaminant is important for understanding the adsorption mechanisms. Hence, in this study, the single contaminant adsorption is examined not only at a neutral pH but also at pH = 4 and pH = 10 using buffer solutions, aiming at observing and understanding possible differences in the adsorption of each pollutant.

In an approach to reach even more realistic setups, mixtures of all chosen contaminants are used as well to study possible adsorption competition onto the AC surface. These experiments were carried out using d.i. water and tap water, providing initial insights into possible interferences with other ions found in water.

## Experimental Section

### Materials

Spent coffee (SC) was obtained from DEK Deutsche Extrakt Kaffee GmbH. MgCO_3_ (light, Carl Roth GmbH), hydrochloric acid (37%, Carl Roth GmbH + Co. KG), methyl orange (100%, Merck KGaA), methylene blue (100%, AppliChem GmbH), diclofenac sodium salt (98%, abcr), tetracycline (Santa Cruz Biotechnology, Inc.), bisphenol A (98%, Sigma-Aldrich), magnesium hydroxide carbonate powder (40–45% MgO, VWR), and pH buffer solutions for pH 4 (citric acid, NaOH, and NaCl) and pH 10 (H_3_BO_3_, NaOH, and KCl) (both Carl Roth GmbH + Co. KG) were used as received.

### Sample Preparation

Dry SC and MgCO_3_ were added to a container at a weight ratio of 1:1, vigorously shaken until homogeneous, and pyrolyzed at 850 °C for 1 h under a static argon atmosphere. Afterward, the resulting black solids were washed with ca. 2 M HCl to remove all Mg mineral residues until the solution became acidic, followed by washing and neutralization with d.i. water and drying at 80 °C. The resulting material will be called IB2001 in the following.

### Material Characterization

Infrared spectroscopy was conducted on a Nicolet iS5 (Thermo Scientific) equipped with an iD7 ATR unit with a diamond crystal, a resolution of 4 cm^–1^, and 32 scans per measurement from 400 to 4000 cm^–1^.

Scanning electron microscopy (SEM) was performed on a JEOL JSM-6510 SEM operated at 15 kV. Prior to imaging, all samples were sputter-coated with Au/Pd for 75 s at 18 mA by using an SC7620 mini sputter coater (Quorum Technologies).

X-ray powder diffraction data were collected on a PANalytical Empyrean powder X-ray diffractometer in a Bragg–Brentano geometry, equipped with a PIXcel1D detector using Cu Kα radiation (λ = 1.5419 Å) operating at 40 kV and 40 mA. θ/θ scans were run from 4° to 70° 2θ with a step size of 0.0131° and a sample rotation time of 1 s. The diffractometer was configured with a programmable divergence and antiscatter slit and a large Ni-beta filter. The detector was set to continuous mode with an active length of 3.0061°.

Raman measurements were performed using a WITec alpha300 Raman microscope with 633 nm laser excitation and a 10× objective (NA 0.25) with a laser power of 1 mW. The spectral integration time was set to 2 s for 10 accumulations. The IB2001 powder sample was flattened on a cleaned glass slide before taking it to the Raman microscope. The baseline correction was performed with the WITec Project 5.1 software. The spectrometer was calibrated with a single crystalline Si wafer, which has a characteristic peak at 520.70 cm^–1^.

The chemical composition and chemical states of the NC were obtained with an Axis Supra+ (Kratos Analytical, UK) X-ray photoelectron spectroscopy (XPS) setup using monochromatized Al Kα radiation for excitation (15 kV, typically 20 mA). CasaXPS software was used for data processing and interpretation. XPS signals were fitted using GL (30) line shapes, combining Gaussian (70%), Lorentzian (30%), and asymmetric Lorentzian line shapes (LA (1.2, 2.5, 5)).^36,37^

The specific surface area and pore structure of the material were determined via nitrogen sorption at 77 K with a BELSORP Max (Microtrac Retsch GmbH). Prior to measurement, each sample was degassed to about 2 Pa at 573.15 K for 3 h. The specific surface area (SSA) was calculated using the Brunauer–Emmett–Teller (BET) method. The average pore size and pore volume were estimated from the adsorption branch of the isotherm using the Barrett–Joyner–Halenda (BJH) method for mesopores. Pore size distributions were calculated using nonlocalized density functional theory (NLDFT) with Tikhonov regularization, assuming slit-shaped pores in the material. All calculations were carried out using the software package BELMaster provided by Microtrac Retsch GmbH.

To determine the point of zero charge (PZC), a 0.01 M aqueous NaCl solution was prepared. Afterward, 10 mL of the solution was adjusted to a starting pH_0_ of 3, 5, 7, 9, and 10 using 0.1 M HCl and 0.1 M NaOH. To these solutions, 50 mg of IB2001 were added, the vials were closed, and agitated at 450 rpm on a shaker. After 24 h, the final pH_e_ of each solution was measured again. The difference ΔpH = pH_0_ – pH_e_ was plotted vs pH_0_. The PZC was reached when ΔpH = 0.

### Surface Area Calculations of Pollutants

For surface area calculations, models of the contaminant molecules were drawn using ChemSketch. Afterward, a combined optimization and frequency calculation was carried out to find the local minimum of each structure using Gaussian 16 Version C01, at the B3LYP-D3/aug-cc-pVDZ level, using a polarizable continuum model for water.^38^ The surface areas were then calculated using Jmol software. The solvent probe radius was set to 1.2 Å. Via the commands “isosurface sasurface” and “isosurface area set 0”, the solvent-accessible surface areas (with and without counterions) of each optimized pollutant molecule were calculated.

### Adsorption Studies

All adsorption experiments were carried out three times for reproducibility. The arithmetic average was then calculated, including the standard deviation, for graphical display.

Methylene blue (MB), methyl orange (MO), diclofenac sodium salt (DCF), tetracycline (TET), and bisphenol A (BPA) were chosen as model contaminants. Starting solutions (*c*_i_ = 200 mg/L) of all compounds were prepared using deionized water (d.i. water), pH4-buffer solution (citric acid, sodium chloride, and sodium hydroxide), and pH10-buffer solution (boric acid, sodium hydroxide, and potassium chloride). For TET and BPA, 10% EtOH was added for better solubility. Between 1 and 50 mg of IB2001 were dispersed in 10 mL of each contaminant solution and shaken on a Heidolph Vibramax 100 shaker at 450 rpm for 1 h at room temperature.

After the termination of the experiments, all solutions were filtered through PTFE syringe filters (VWR, 0.45 μm) and examined via UV/vis spectroscopy between 200 and 1000 nm. The uptake of contaminants by the adsorbents was determined ratiometrically using the intensity of the absorption bands (MO @ 461 nm, MB @ 664 nm, DCF @ 276 nm, BPA @ 225 nm, and TET @ 358 nm) after adsorption, using calibration curves measured beforehand for each component. Depending on the pH, these bands can be slightly shifted. In these cases, the evaluation was adjusted accordingly.

Additionally, a mixture of all contaminants was prepared in d.i. water as well as in laboratory tap water. The pH of the d.i. water was 6.77, and that of the tap water was 8.24. For this part of the study, the initial concentrations *c*_i_ of each contaminant were 50 mg/L, dissolved in 10% EtOH. Of these solutions, 10 mL were shaken with 50 mg of IB2001 for 1 h and then filtered. Because of overlapping adsorption bands in UV/vis, the resulting solutions were analyzed via HPLC-MS.

UV/vis spectroscopy was performed on a Shimadzu UV-1900, between 200 and 1000 nm, with a sampling interval of 1.0 nm at medium speed in single scan mode. For TET, DCF, and BPA, quartz cuvettes were used. The spectra of the dyes MB and MO were measured by using PMMA cuvettes. All cuvettes had a path length of 1 cm.

HPLC-MS was carried out on an Agilent Infinity 1260 System using a Column Kinetex C8, 2.6 μm, 100 Å, 150 × 4.6 mm (Phenomenex, Torrance, CA, USA), at a column temperature of 30 °C and a flow rate of 0.5 mL/min. Eluents were 0.1% formic acid and acetonitrile (Merck, Darmstadt, Germany). The sample volume was set at 20 μL at a sampling temperature of 10 °C. The HPLC was coupled with an Agilent G6470A Series Triple Quad LC/MS (Agilent Technologies Sales and Services GmbH and Co. KG, Waldbronn, Germany).

### Adsorption Isotherms

The absolute percentage of removal *R* of a contaminant is always one of the first values of interest in adsorption studies, using the initial concentration of contaminant *c*_i_ and the equilibrium concentration after adsorption *c*_e_. *R* is defined as follows (eq 1):
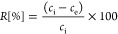
1

Using the UV/vis absorption data, the equilibrium concentrations *c*_e_ were calculated for each batch and plotted using the calibration data previously reported.^35,39^ With the initial and equilibrium concentrations, the amount of adsorbate at equilibrium *q*_e_ in mg/g was calculated with the mass of adsorbent *m* and volume of solution *V* (eq 2).
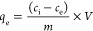
2

The Langmuir (eq 3) and Freundlich models (eq 4) were applied to gain insight into the adsorption mechanisms.^40,41^ While the Langmuir model assumes the formation of a monolayer on homogeneous surfaces, Freundlich isotherms assume multilayer adsorption onto heterogeneous surfaces.^6,9,42^*Q*_sat_ describes the adsorption capacity that is given when the monolayer on the adsorbent is fully covered, *K*_L_ is the Langmuir equilibrium constant. In the Freundlich equation, *K*_F_ is called the Freundlich constant, and 1/*n* describes the surface heterogeneity.^42^
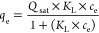
3
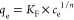
4

## Results and Discussion

### Material Characterization

**Figure 1:**
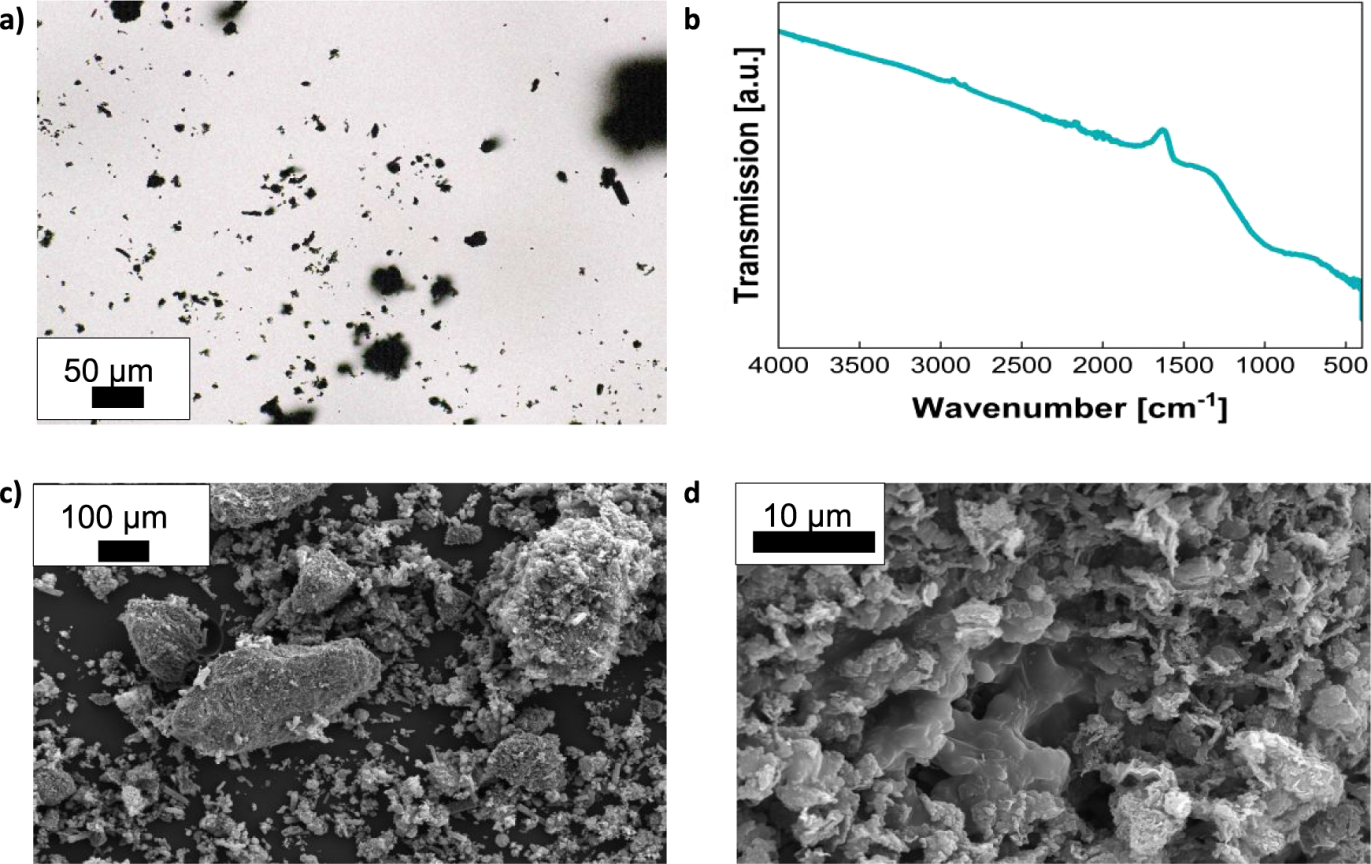
(a) Optical microscopy image of washed and dried IB2001, (b) IR spectrum and (c,d) electron microscopy images of IB2001 at different magnifications after washing before adsorption processes.

Pyrolysis with MgCO_3_ and subsequent washing with HCl of the SC result in the formation of a very fine powdered carbon material, hereinafter called IB2001. Optical and scanning electron microscopy show particles of different sizes and shapes, as well as larger agglomerated pieces with pitted and uneven surfaces similar to earlier generations of the material (Figure 1a,c,d).^35^ IR spectroscopy shows no bands of any functional groups in the material (Figure 1b).

**Figure 2:**
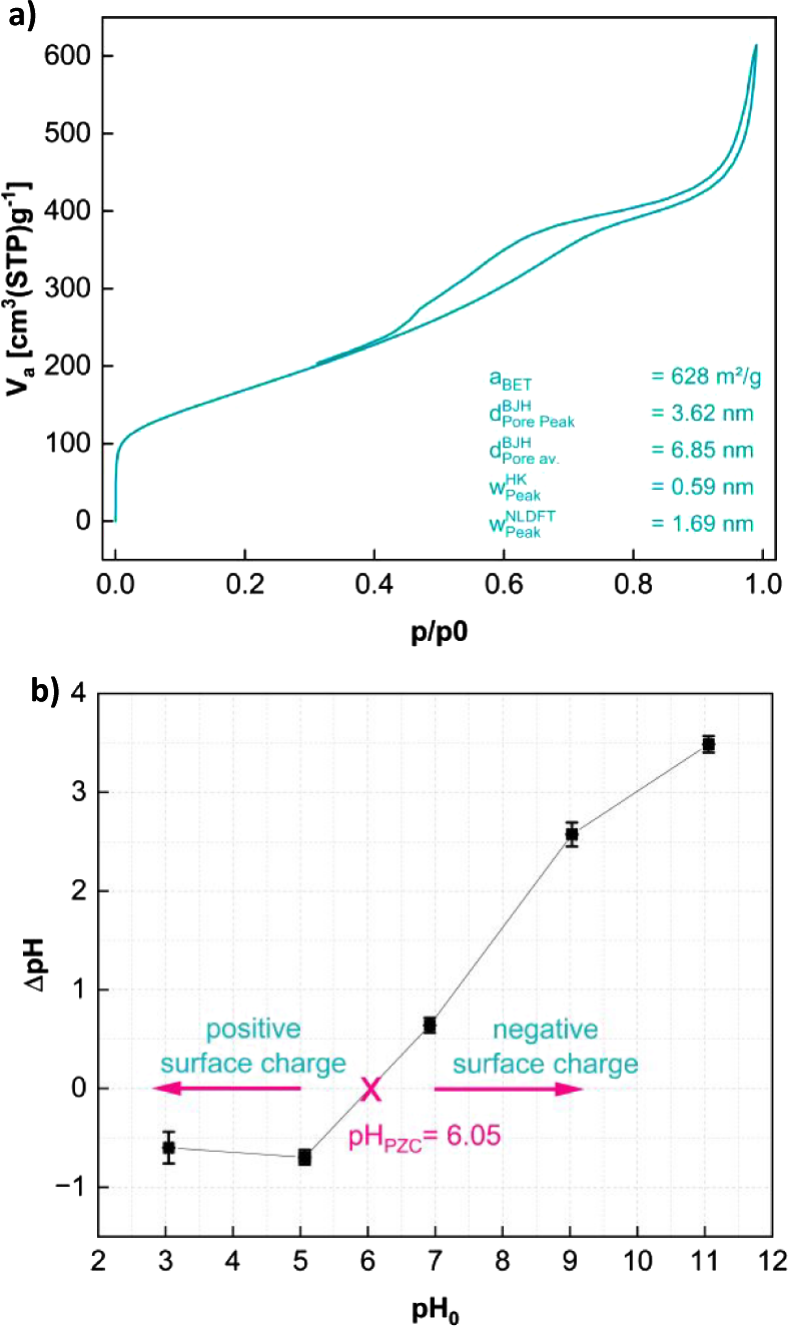
(a) N_2_ sorption isotherm and (b) point of zero charge measurement of IB2001.

The nitrogen sorption isotherm (Figure 2a) shows a continuous slope with a small plateau between *p*/*p*_0_ = 0.7 and 0.9. BET analysis of the data results in an average surface area (*a*_BET_) of 628 m^2^/g (arithmetic mean of 5 measurements). This is in the higher range of surface areas for biochars, especially considering that no chemical pretreatment was executed. Also, it is over three times larger than a material published previously, where CaCO_3_ was used as an additive instead of MgCO_3_.^35^

IB2001 contains a mixture of pore types (mesopores and some micropores), as determined from the N_2_ adsorption isotherms. According to the BJH evaluation, the average diameter of the mesopores in the current material, IB2001, is 6.85 nm (), with the main peak at 3.62 nm (). The average width of the micropores in IB2001 is 0.59 nm (*w*_Peak_^HK^). The theoretical approach of the nonlocalized DFT calculations (NLDFT) shows micropores of 1.69 nm (*w*_Peak_^NLDFT^).

The PZC is reached at pH 6.05, as shown in Figure 2b. Hence, at a solution pH < 6.05, the IB2001 biochar particles are positively charged, while at a solution pH > 6.05, the biochar is negatively charged.^20,43,44^

**Figure 3:**
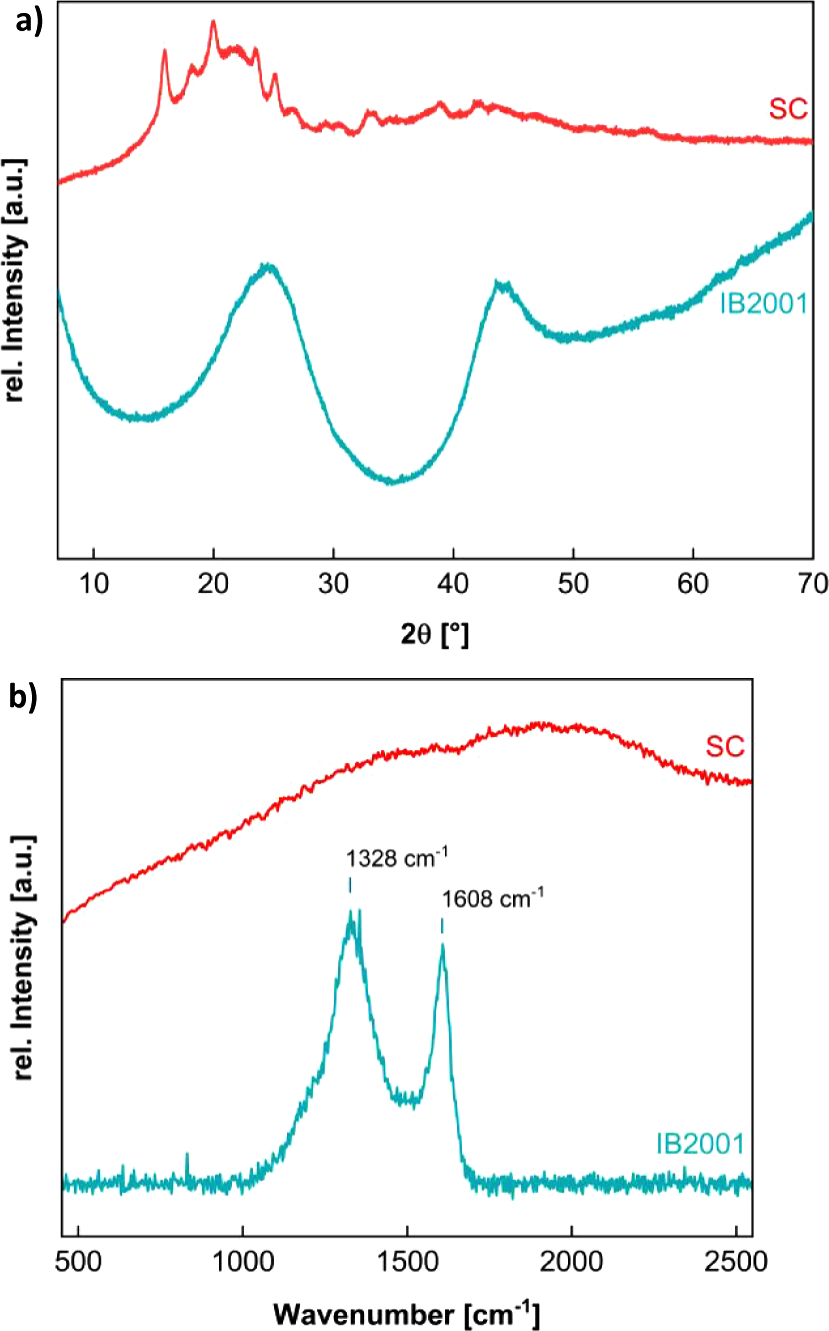
(a) X-ray diffractogram. (b) Raman spectra of raw SC and IB2001.

In X-ray diffractograms, the untreated SC shows a series of reflexes on top of two broader shoulders, which is a common pattern for cellulosic materials.^35,45^ Similar to the data obtained for a different material published in 2021, the X-ray diffractogram (Figure 3a) of IB2001 shows only two very broad reflections at around 25° and 43° for the graphitic carbon planes (002) and (100).^24,35,46^ This may also indicate a parallel stacking of graphene planes, as well as a disordered or even partially material.^47,48^

While the Raman spectrum (Figure 3b) of raw SC does not show any signals to be evaluated, the pyrolyzed sample IB2001 shows two significant, not completely separated bands that can be identified as the D (1328 cm^–1^) and G (1608 cm^–1^) bands of carbon materials. The ratio of peak intensities *I*_D_/*I*_G_ and their full width at half-maximum (fwhm) suggest the quality of the carbon material. Here, the high intensity of the D peak corresponds to a higher number of defects or the presence of many small graphene flakes with numerous edges. Based on the peaks’ ratio and the fwhm of the D peak, the material is primarily nanocrystalline graphite with a significant number of defects. This could also suggest small crystalline domains within the carbon material, where the high surface area to volume ratio results in a high number of edge defects. Such a spectrum suggests lower crystallinity and a higher presence of sp^2^ carbon ring structures disrupted by defects.^30,49−51^

**Figure 4:**
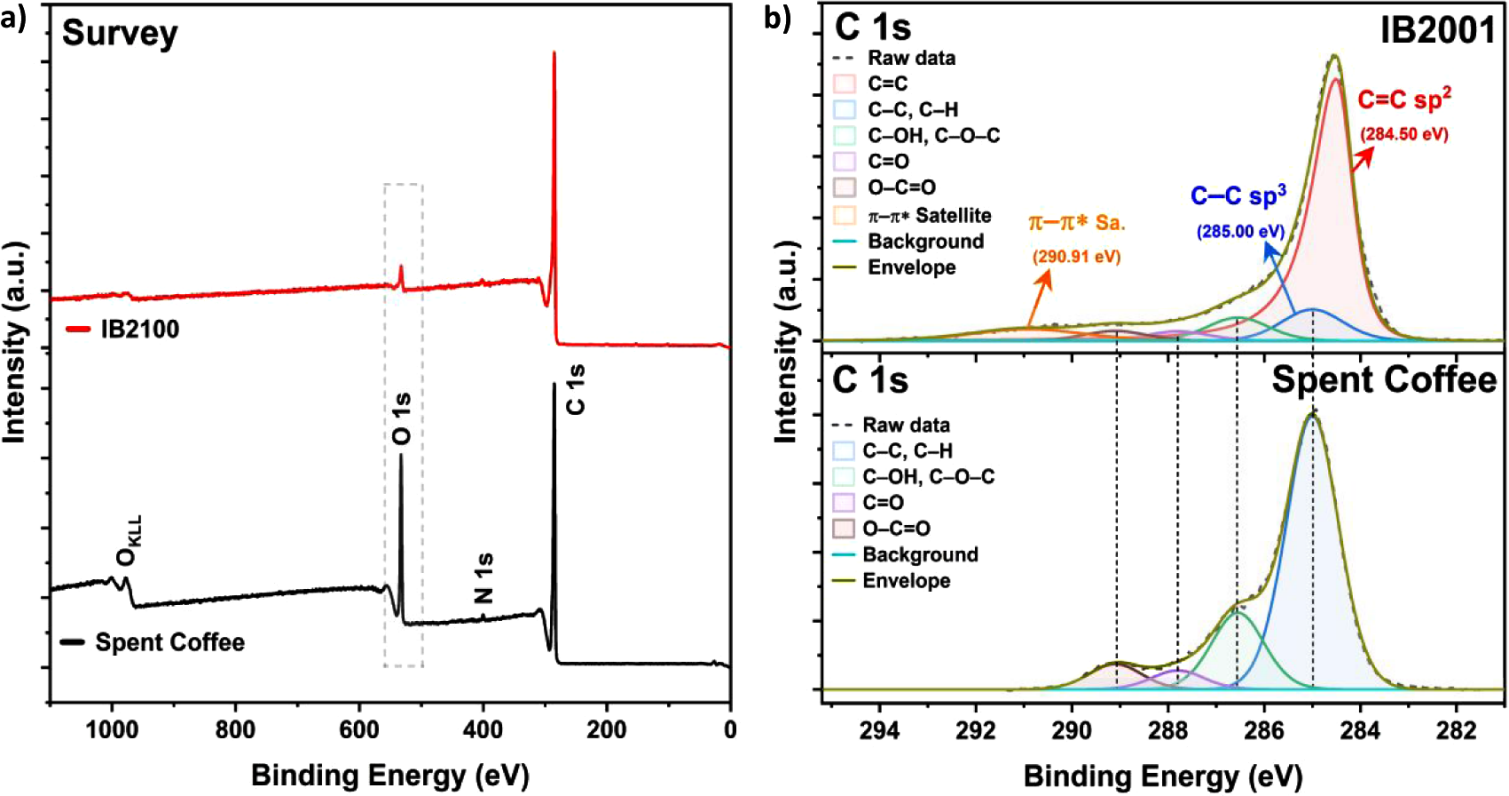
High-resolution XPS spectra of raw SC and IB2001: survey (a) and C 1s (b) spectra, respectively.

XPS analysis was conducted to investigate and compare the chemical state information of the elements present in IB2001 and raw SC samples. In Figure 4a, the survey spectra in the range from 0 to 1100 eV show that both samples contain relatively strong C 1s signals, while the O 1s signal of IB2001 is less visible than in the spectrum of SC. These data suggest a carbonization reaction at a high temperature of 850 °C that may be accompanied by a dehydration reaction, resulting in the formation of double bonds, etherification, and/or esterification.

In Figure 4b, the binding energies of C–C bonds, mainly attributed to sp^3^ hybrid carbons, were charge-corrected by setting them equal to 285.0 eV in both samples, and their oxygen-bound species of C 1s peaks are identified as C–O, C=O, and O—C=O bonds at 286.55, 287.8, and 289.1 eV equally in both samples, respectively.^36,52,53^ Meanwhile, the C 1s of IB2001 mainly contributes to the graphitic (C=C) peak at 284.5 eV compared to its aliphatic (C–C and C–H) peak at 285.0 eV, and its π to π* shakeup satellite is simultaneously detected at 290.91 eV.^36,54^ These data may indicate that IB2001 has characteristics of graphitic materials (e.g., graphite, graphene, and carbon nanotubes) when compared to raw SC.^55,56^ In addition, this partial structural transformation from sp^3^ to sp^2^ is well-matched with the Raman spectra data, where only pyrolyzed IB2001, not raw SC, revealed a G line around 1608 cm^–1^ that can be assigned to the E_2g_ photon of sp^2^ carbons (Figure 3b).^56^

Taking all XRD, IR, and Raman results into account, the material appears rather complex, and we currently interpret the data as an indication of a mixture of glassy carbon with nanocrystalline fractions and a rather high disorder. Additionally, there is likely a rather high number of defects present in this material.

### Adsorption Studies

Batch studies for the determination of the adsorption capacity of IB2001 using TET, BPA, MB, MO, and DCF (Figures S4–S7) were conducted for each contaminant separately. Experiments were carried out in an aqueous solution (d.i. water without adjusting the pH, pH ≈ 6.8) as well as in acidic (pH 4) and basic (pH 10) media using buffer solutions.

All contaminants tested, except TET, had adsorption isotherms that matched the Freundlich model rather than the Langmuir model based on their determination coefficients *R*^2^ (Table 2). This is irrespective of the solution pH or type of contaminant. For TET, the *R*^2^ values are slightly larger for the Langmuir isotherms at all pH values. Given the small differences, this is likely within the experimental error of both analyses. Hence, we can assume a heterogeneous particle surface for IB2001 with multilayer physisorption taking place for all contaminants. Looking at the correlating adsorption data plotted in Figures S4–S7, for all pollutants, adsorption capacities *q*_e_ are well above 100 mg/g. Comparing these values to the materials displayed in Table 1 and considering the high surface area of IB2001, the data clearly show that the current process is highly effective for the preparation of an interesting and promising biochar.

**Table 2:** Adsorption Isotherm Parameters for the Freundlich and Langmuir Models Applied to the Mean of 3 Batch Adsorption Studies Per Contaminant^a^^b^^c^

		Freundlich				Langmuir			
		*K*	*n*	*R*^2^	*R*^2^_korr_	*Q*_sat_	*K*	*R*^2^	*R*^2^_korr_
pH 4	TET	51.1317	3.4332	0.9043	0.8851	160.5296	0.2593	0.9717	0.9660
	MB	60.0625	2.4732	0.8795	0.8554	213.0003	0.2610	0.7315	0.6778
	MO	71.3212	3.5623	0.8337	0.7921	135.6411	1.8114	0.5010	0.3763
pH 6.8	DCF	16.7680	3.0730	0.6369	0.5643	90.4414	0.0452	0.6191	0.5430
	BPA	16.7755	1.8335	0.9339	0.9207	201.9890	0.0410	0.8918	0.8702
	TET	43.2795	3.3919	0.8326	0.7992	142.5445	0.2279	0.8418	0.8102
	MB	64.8090	4.7882	0.8793	0.8491	97.8847	5.5138	0.6921	0.6151
	MO	72.5017	5.0641	0.7673	0.6897	126.6446	2.6351	0.4635	0.2846
pH 10	DCF	32.6390	2.7969	0.9649	0.9579	138.3175	0.1524	0.8548	0.8258
	BPA	20.4889	1.9346	0.9884	0.9846	224.3796	0.0401	0.9510	0.9347
	TET	25.8629	2.7844	0.9164	0.8997	137.7119	0.0745	0.9327	0.9193
	MB	98.4614	3.8914	0.9485	0.9382	184.5953	1.3957	0.7735	0.7282
	MO	64.4104	4.1448	0.9160	0.8991	132.3469	1.2817	0.7247	0.6700

a For DCF and BPA, no adsorption study was carried out at pH 4 due to precipitation of the contaminant in solution.

b *R*^2^ is the determination coefficient.

c The correlating data sets and plots are given in Figures S4–S7.

For DCF and BPA, no adsorption isotherms could be determined at pH 4. DCF shows little to no solubility in the buffer solution, while BPA showed significant noise around both absorption bands at 225 and 276 nm, preventing a reasonable analysis of these data sets. These problems do not occur for all other contaminants examined or at other pH values.

**Figure 5:**
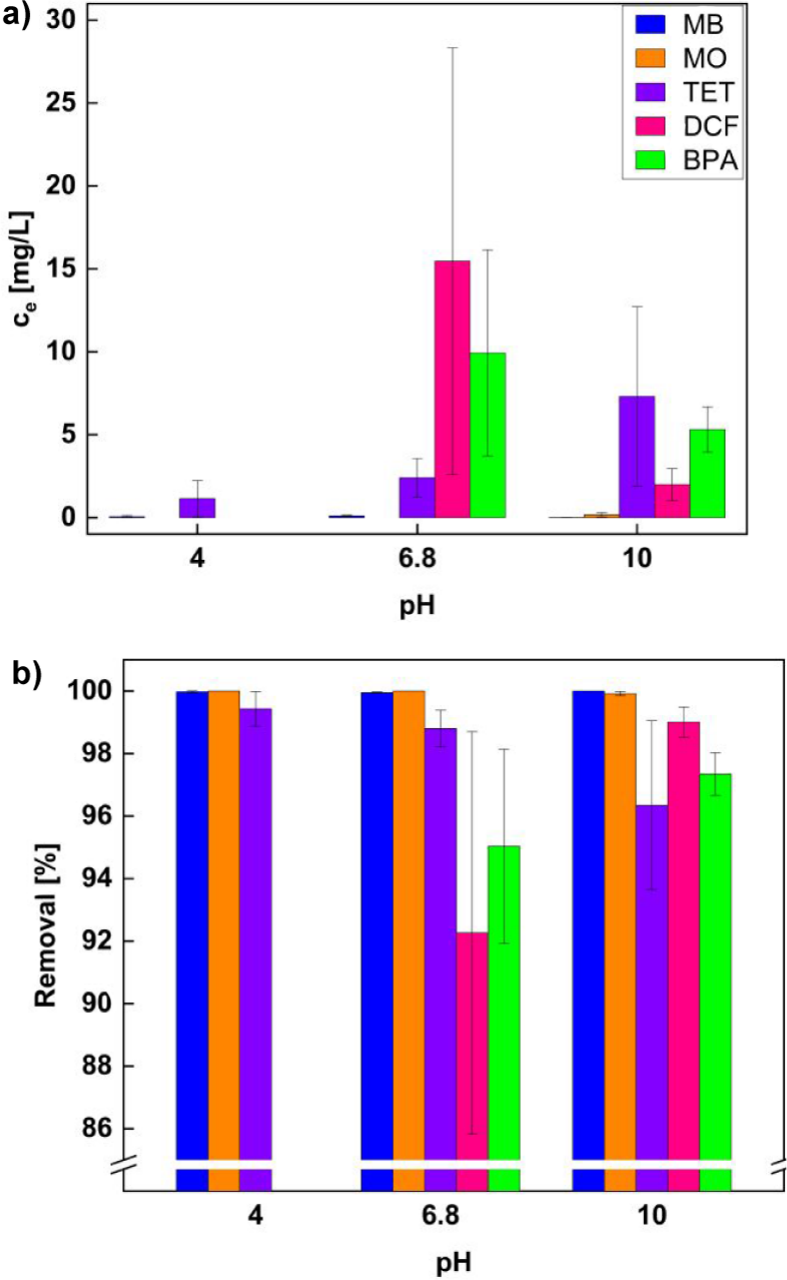
(a) Remaining concentration and (b) absolute removal percentage of contaminants after 1 h of adsorption onto 50 mg of IB2001 at different pH values. Starting concentrations were 200 mg/L each, *n* = 3, error bars show standard deviation of *c*_e_ and removal percentage. An alternative presentation of a) is given in Figure S3.

Overall, all contaminants show low remaining concentrations *c*_e_ in the solutions after treatment with 50 mg of IB2001 biochar (Figure 5a) and very high removal percentages of >90% in UV/vis measurements (Figure 5b) with a starting pollutant concentration of 200 mg/L each. While the dyes MO and MB are nearly completely removed at all pH values, TET, DCF, and BPA show slight pH dependency. TET is most efficiently removed in acidic media (99.4%) and least effectively in basic media (96.3%) with a higher error margin. On the other hand, BPA and especially DCF are best removed under basic conditions (97.3% for BPA and 99.0% for DCF) with smaller standard deviations than in roughly neutral (pH = 6.8) aqueous media (95.0% for BPA and 92.2% for DCF). However, when one looks at the error bars (Figure 5a), these differences are, within the error margin, essentially identical.

To explain these differences, calculations of the surface areas of the contaminant molecules (with and without counterions) using the software Jmol were carried out (Table 3). The results show no direct connection between the molecular surface area and adsorption behavior. For example, TET is neutral and has the largest surface area of all the molecules investigated here. It is absorbed somewhat less effectively on IB2001 than on MB and MO, both of which have a smaller surface area. This could indicate that surface area is more important than charge, but again, as a selected example, TET exhibits a higher removal rate in acidic media than in basic media, while BPA and DCF show the opposite trend. These molecules have a smaller surface area, but the charge distribution is different in all of them. For example, BPA is uncharged, yet it is adsorbed more effectively than DCF. Overall, these data show that there is no direct nor monocausal explanation for the exact behavior of each of the compounds, much less so when they are in a competitive environment like the mixed solutions studied here (see below for details).

**Table 3:** Molecular Weight and Calculated Solvent Accessible Surface Areas of Contaminants Setting Water as a Solvent Using Jmol^a^

	*M* [g/mol]	Surface area [Å^2^]	Surface area [Å^2^] including counterions
BPA	228.29	400.1	-
DCF	319.14	423.1	455.9
TET	444.44	557.9	-
MB	319.85	477.9	513.6
MO	328.34	498.3	528.8

a BPA and TET as uncharged molecules do not have counterions; hence, no data for these species can be given.

**Figure 6:**
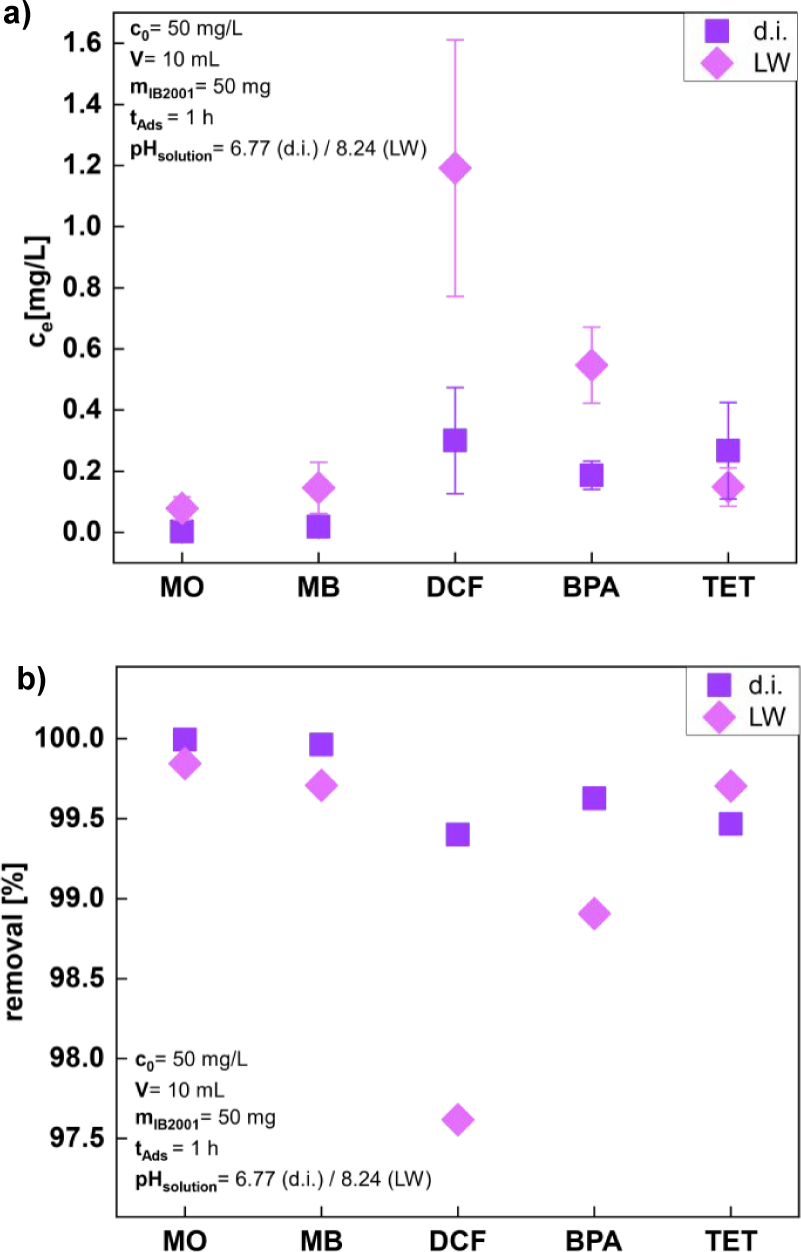
(a) Equilibrium concentration *c*_e_ of each contaminant (*c*_0_= 50 mg/L, *V* = 10 mL) after a 1 h of simultaneous adsorption on 50 mg of IB2001 in d.i. water (d.i.) and laboratory tap water (LW) and (b) the absolute removal percentage of each contamination. Note that the scale in panel b is strongly expanded only showing the region from 97 to 100%.

We have outlined a few of the challenges with respect to the identification of the effects in single-contaminant model solutions. These data show that even these systems are rather challenging in terms of exact model development. These experiments, however, do not represent realistic conditions for water treatment. Therefore, to provide a more realistic impression of the IB2001 material, additional experiments were conducted using a mixture of all contaminants in either deionized water (d.i.) or laboratory tap water (LW, Figure 6). Due to the miscibility of contaminants for these experiments, the concentration of contaminants was lowered to 50 mg/L each.

In d.i. water, the dyes MB and MO are adsorbed best. HPLC-MS measurements show remaining concentrations for both dyes are below the limit of quantification (LOQ, Table 4), meaning a removal rate of nearly 100%. DCF and TET show a similar removal of about 99.5%, BPA is slightly better removed, but not completely, as seen in Figure 6a,b.

**Table 4:** Remaining Concentrations as Numerical Values of Contaminants in Aqueous Mixtures After Treatment with IB2001 Determined Using HPLC-MS

		MO	MB	BPA	DCF	TET
*c*_e_ [mg/L]	d.i.	LOQ 0.0027 (±0.0013)	LOQ 0.0181 (±0.0081)	0.1867 (±0.0462)	0.3000 (±0.1734)	0.2667 (±0.1582)
	LW	0.0779 (±0.0380)	0.1455 (±0.0840)	0.5467 (±0.1242)	1.1916 (±0.4200)	0.1483 (±0.0625)

In tap water, a similar trend can be observed. Overall, the contaminants are removed by over 97%. However, it is to be noted that the initial concentrations of each contaminant were a quarter of those in the single-contaminant setups described above.

As IR, XRD, and XPS have shown beforehand, IB2001 has few to no functional groups on the particle surface. Thus, it can be assumed that physisorption is the dominant driving force for adsorption in all systems. Typical causes for different adsorption behavior may be differences in charges, charge distribution, formation of hydrogen bonds, or different strengths or types of van der Waals interactions, etc.

As discussed above for the single-contaminant systems, calculations of surface areas (with and without counterions) of the pollutants (Table 3) show no direct connection between surface area and adsorption properties. This also suggests that a multitude of different and possibly competing interactions are responsible for the observed behavior.

To further complicate the situation, some contaminants show a rather direct interaction with components present in LW but not in d.i. water. For example, the behavior of DCF in d.i. water compared to DCF in tap water may be explained by the chelation of metal cations (M^2+^, likely Ca^2+^ and/or Mg^2+^ ions) by DCF.^57,58^ This effect is already observed when dissolving the contaminants in LW. The solution prepared with LW is not perfectly clear but slightly cloudy, while in d.i. water, a completely clear solution is always obtained. Clearly, in real systems, the presence of metal cations cannot be excluded, and additional effects can therefore be expected for more complex, realistic water matrices. In spite of this, the IB2001 material is highly effective for a number of quite diverse (micro)pollutants.

## Conclusion

The surface area and porosity of biochars are among the most important parameters for tuning biochars to a certain application – generally, the larger the surface area and the more accessible the pores, the more attractive a biochar is for a variety of applications. The current report describes a simple yet highly effective approach toward an improved biochar from spent coffee: replacing CaCO_3_ with MgCO_3_ as a pore-former leads to an enlargement of the surface area of the biochar particles to over 600 m^2^/g, which is quite striking for biochars. The large surface area leads to highly improved physisorption, following the Freundlich model of multilayers in single contaminant systems. Further characterization methods, such as XRD, XPS, and Raman spectroscopy, support the hypothesis of a fully carbonaceous material mostly without functional groups on its surface area, allowing only physisorption to take place. IB2001 works exceptionally well for a range of quite diverse organic contaminants and under a wide range of conditions. For some pollutants, apparent trends in adsorption considering the pH value of the solutions are observed. For example, the antibiotic TET is better adsorbed in acidic media, while the pharmaceutical DCF tends to better adsorption at higher pH. Even when mixed solutions are employed, the IB2001 biochar removes over 97% of the contaminants from solutions prepared from tap water. In contaminant mixtures in tap water, DCF is the least adsorbed. This is possibly due to complexation and chelation. However, these trends and preferences in adsorption behavior can only partially be explained, despite extensive characterizations so far. In spite of this, considering the high surface area and the excellent adsorption capacities, IB2001 is a highly promising biochar from renewable and widely accessible raw materials.

## Data Availability

The data underlying this study are not publicly available due to connections to a number of manuscripts in preparation. The data are available from the corresponding authors upon reasonable request via email.

## Supporting Information Available

ATR-IR spectrum of spent coffee grounds (Figure S1); high-resolution XPS spectra of O 1s in IB2001 and raw SC (Figure S2); alternative display of remaining concentrations of contaminants at different pH values (Figure S3); adsorption isotherms of BPA and DCF in d.i. water and at pH = 10 with modeled Langmuir and Freundlich isotherms (Figure S4); adsorption isotherms of TET in d.i. water and at pH = 4 and pH = 10 with modeled Langmuir and Freundlich isotherms (Figure S5); adsorption isotherms of MB in d.i. water and at pH = 4 and pH = 10 with modeled Langmuir and Freundlich isotherms (Figure S6); adsorption isotherms of MO in d.i. water and at pH = 4 and pH = 10 with modeled Langmuir and Freundlich isotherms (Figure S7) (PDF)
