# Supplementary material for: Improving Spent Coffee Biochar for Effective Organic Contaminant Removal from Aqueous Media
Source: ACS Omega. 2025 Jan 30;10(5):4614–23. doi: 10.1021/acsomega.4c09171 (PMC11822482; doi:10.1021/acsomega.4c09171)
Supplement: Supplementary file 1 — ao4c09171_si_001.pdf [file ao4c09171_si_001.pdf]

Supporting Information
Improving spent coffee biochar for effective organic contaminant removal from aqueous media

Inga Block[1,*], Harshadrai M. Rawel[2], Tillmann Klamroth[1], Christina Günter[3], Jiyong Kim[4], Fabian Loepthien[4], Shashank K. Gahlaut[1], Ilko Bald[1], Andreas Taubert[1,*]

1 Institute of Chemistry, University of Potsdam, Karl-Liebknecht-Straße 24-25, D-14476 Potsdam, Germany
2 Institute of Nutritional Science, University of Potsdam, Arthur-Scheunert-Allee 114-116, D-14558 Nuthetal, Germany
3 Institute of Geosciences, University of Potsdam, Karl-Liebknecht-Straße 24-25, D-14476 Potsdam
4 Fraunhofer Institute for Applied Polymer Research (IAP), Geiselbergstrasse 69, D-14476 Potsdam, Germany

*iblock@uni-potsdam.de
*ataubert@uni-potsdam.de; www.taubert-lab.net

ATR-IR-Spectroscopy

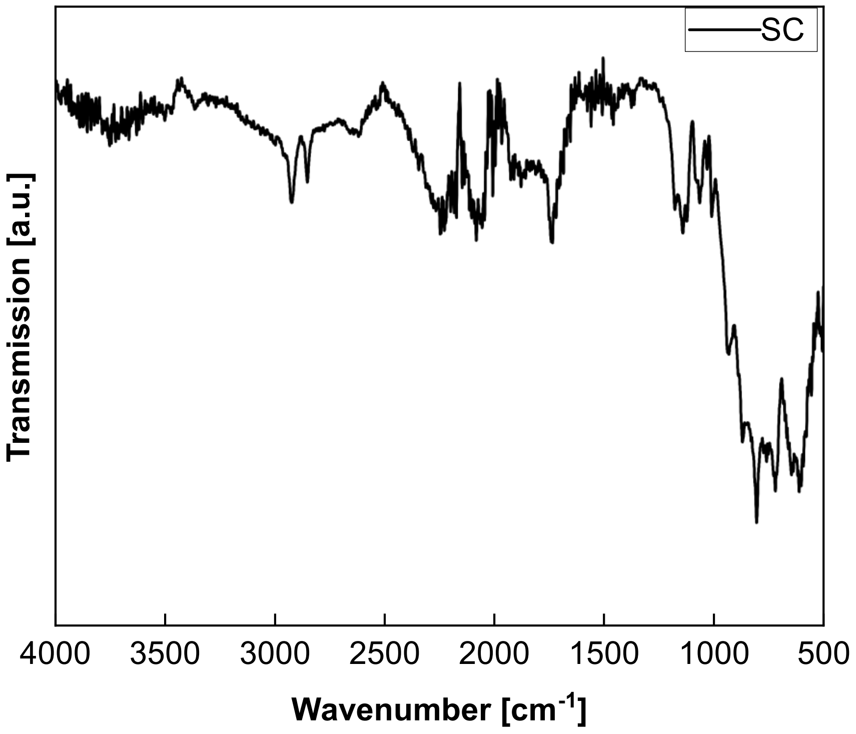

Figure S1 : ATR-IR spectrum of raw and non-pyrolized spent coffee as published in Block et al. (2021)[1]

Before pyrolysis SC shows several IR bands (Figure S1) belonging to different functional groups on the particles surface from its aromatic compounds, residues of oils, caffeine, cellulose and other polysaccharides. All of these signals disappear during the pyrolysis process as shown in Figure 1b in the manuscript.

X-ray photoelectron spectroscopy

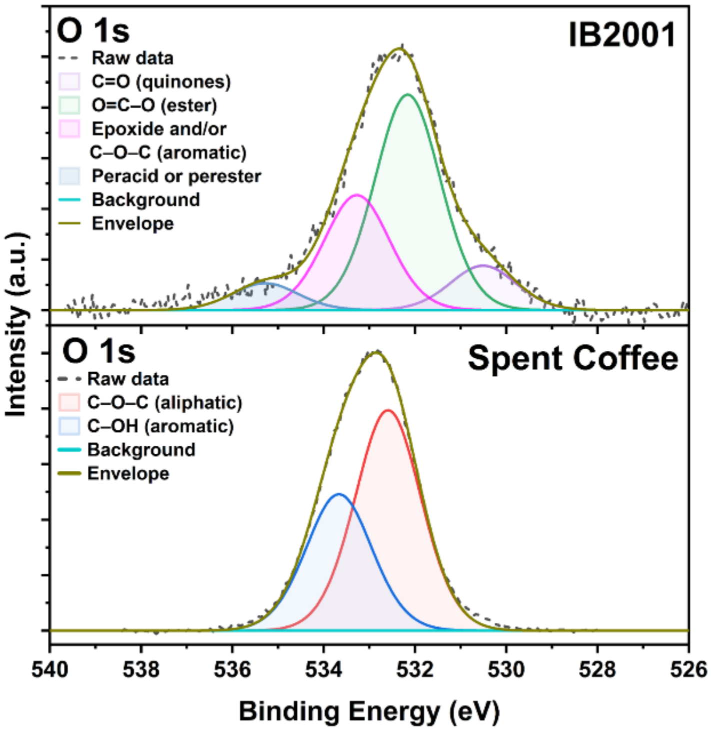

Figure S2 High-resolution XPS spectra of O 1s in IB2001 and raw SC.

Figure S2 shows photoelectron signals from O 1s of IB2001 and spent coffee in O 1s for contrast. The O 1s of spent coffee revealed the general forms of aliphatic ether and aromatic alcohol at 532.59 eV and 533.67 eV, respectively. [2] After carbonization process at high temperature, the IB2001's O 1s signal was well-fitted as C=O (quinones) and O=C–O (ester) bonds at 530.51 eV and 532.16 eV, respectively. [2-4] This result can indirectly support the survey data that the spent coffee undergoes a dehydration reaction while forming graphitic carbons during the carbonization process at high temperatures. Additionally, C–O–C (aromatic) and peracid (and/or perester) bonds were revealed at 533.27 eV and 535.28 eV, respectively, which alludes that harsh reaction conditions at high temperatures under the carbonization process can induce the formation of an oxidation state on the surface of sp^2 hybrid graphitic carbons.[2,5]

Adsorption studies

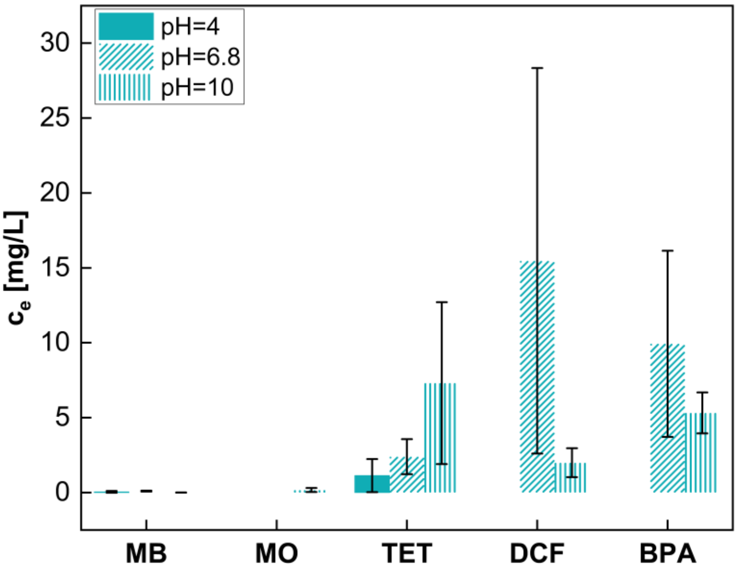

Figure S3: Remaining concentration of contaminants after 1 hour of adsorption onto 50 mg IB2001 at different pH values. Starting concentrations were 200 mg/L each, n=3, error bars show standard deviation of c_e. (Alternative display)

Adsorption isotherms

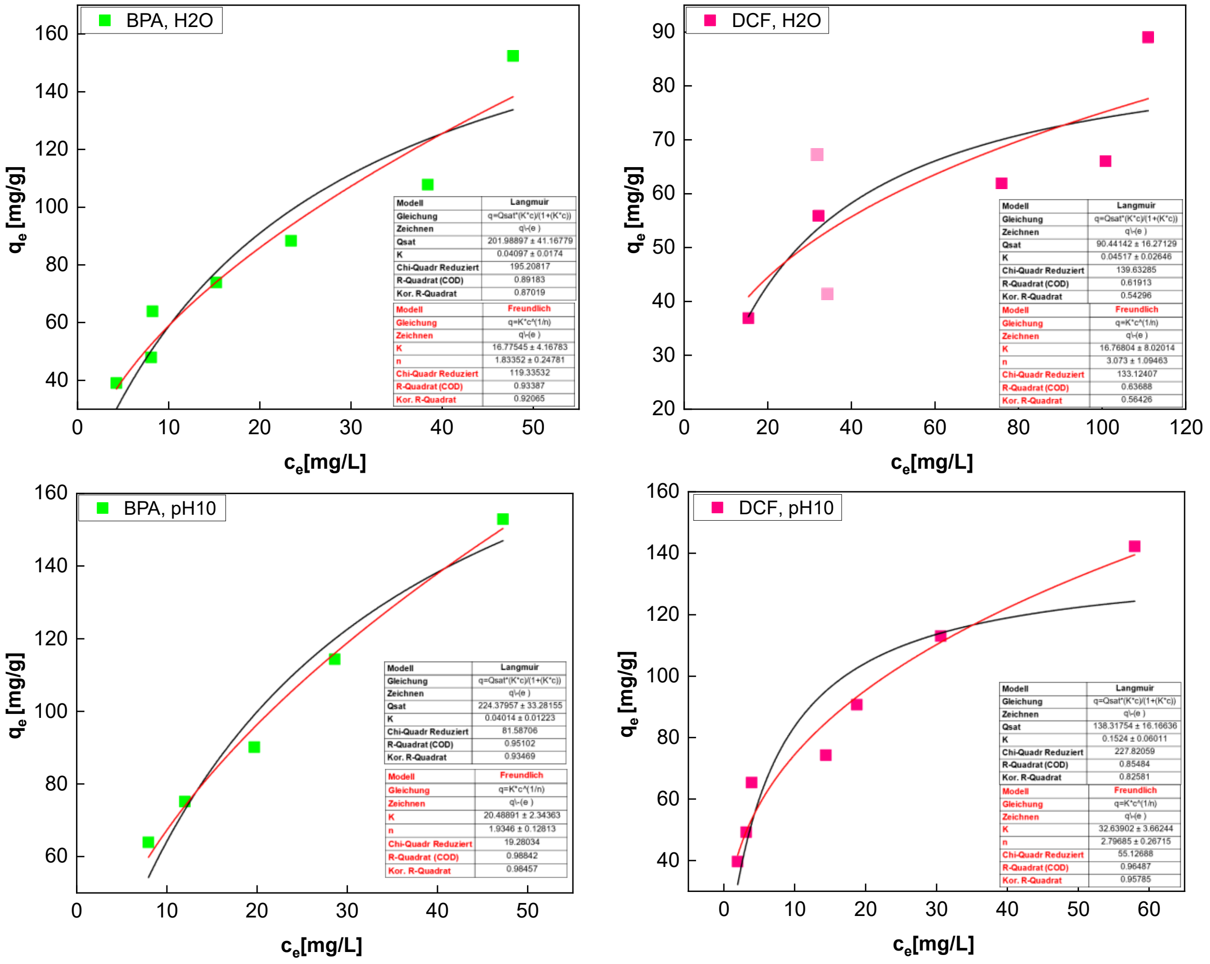

Figure S4: Adsorptions isotherms of BPA (left) and DCF (right) in d.i. water (top) and at pH10 (bottom) including the modelled Langmuir and Freundlich isotherms.

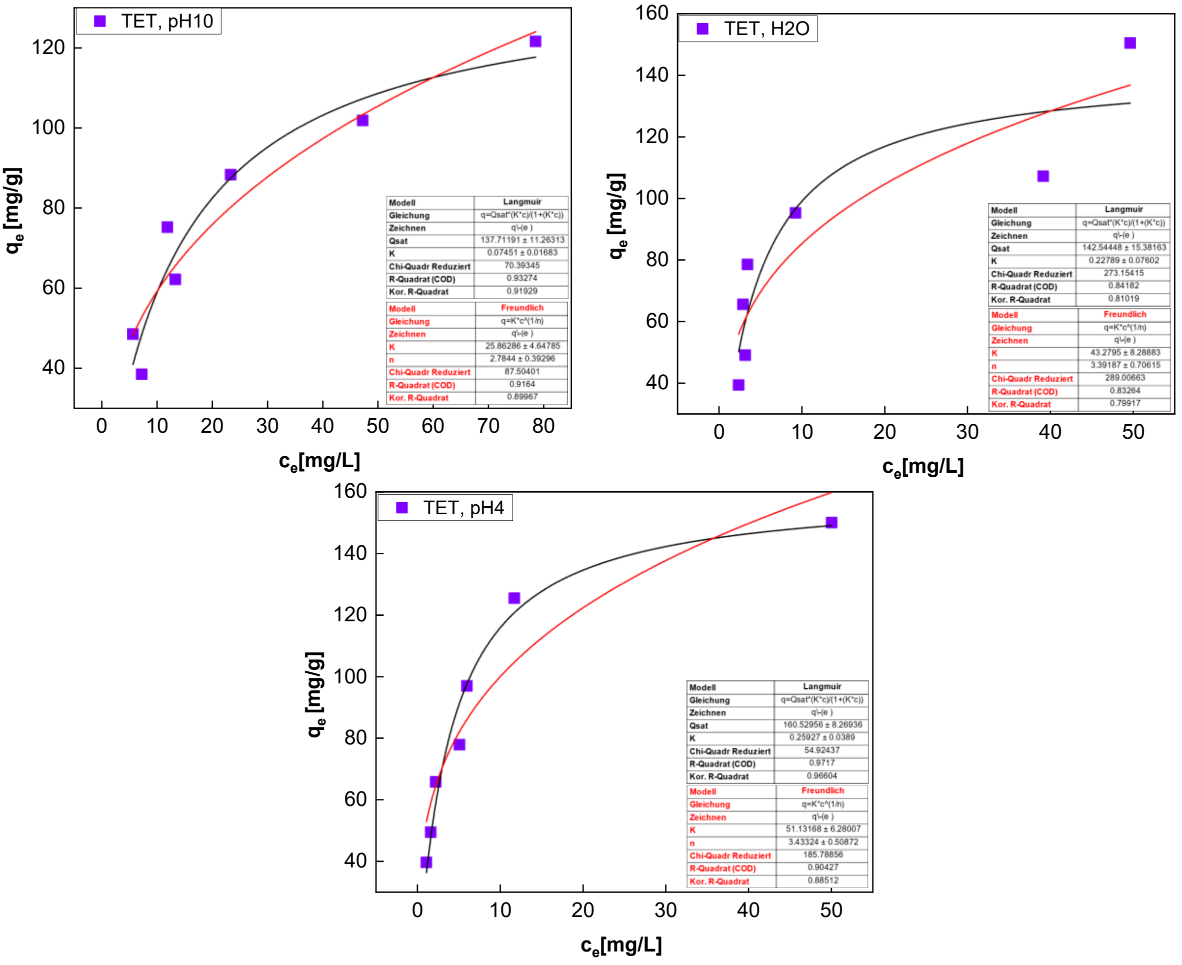

Figure S5: Adsorptions isotherms of TET at pH4 (top left), in d.i. water (top right) at pH10 (bottom) including the modelled Langmuir and Freundlich isotherms

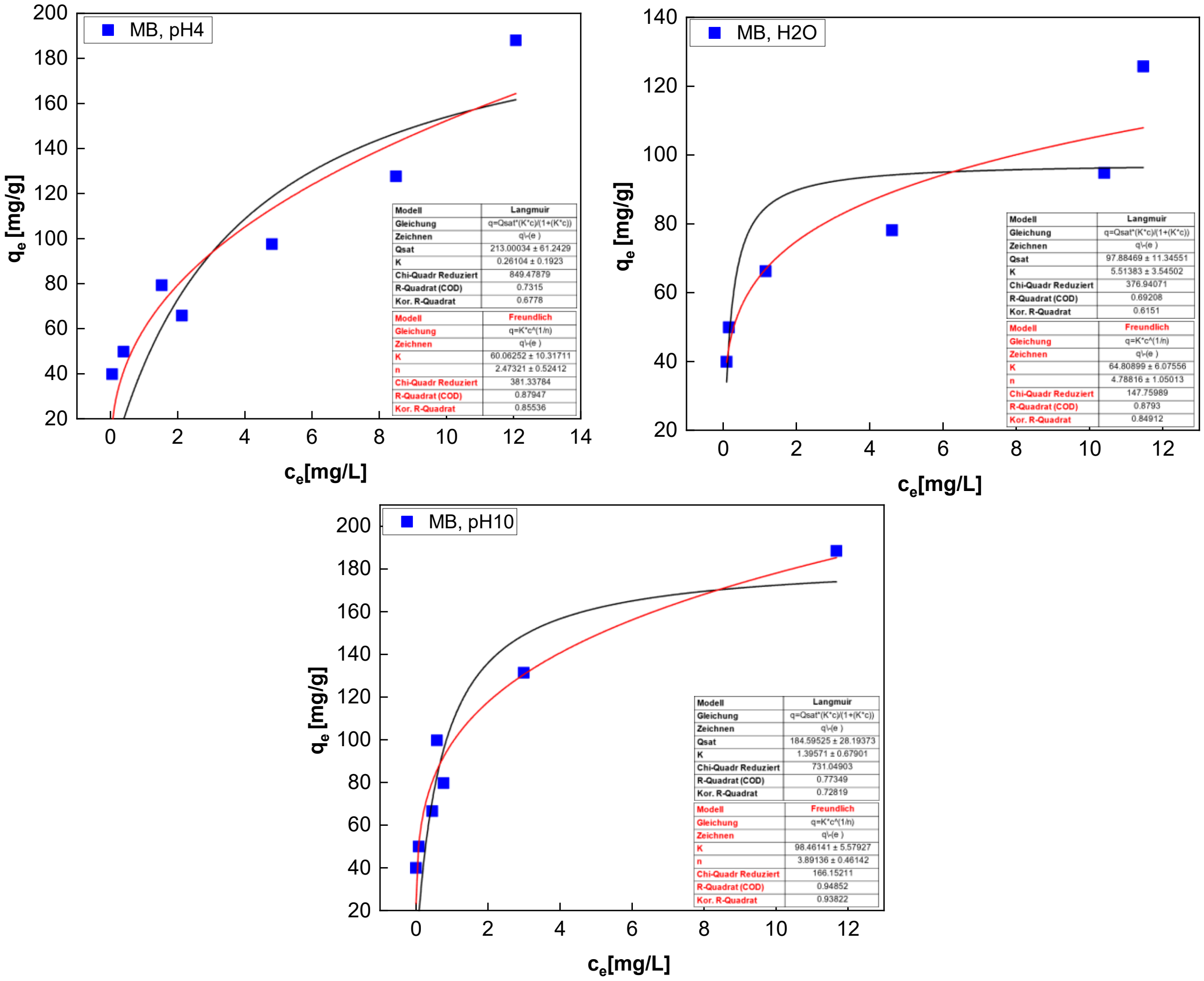

Figure S6: Adsorptions isotherms of MB at pH4 (top left), in d.i. water (top right) at pH10 (bottom) including the modelled Langmuir and Freundlich isotherms

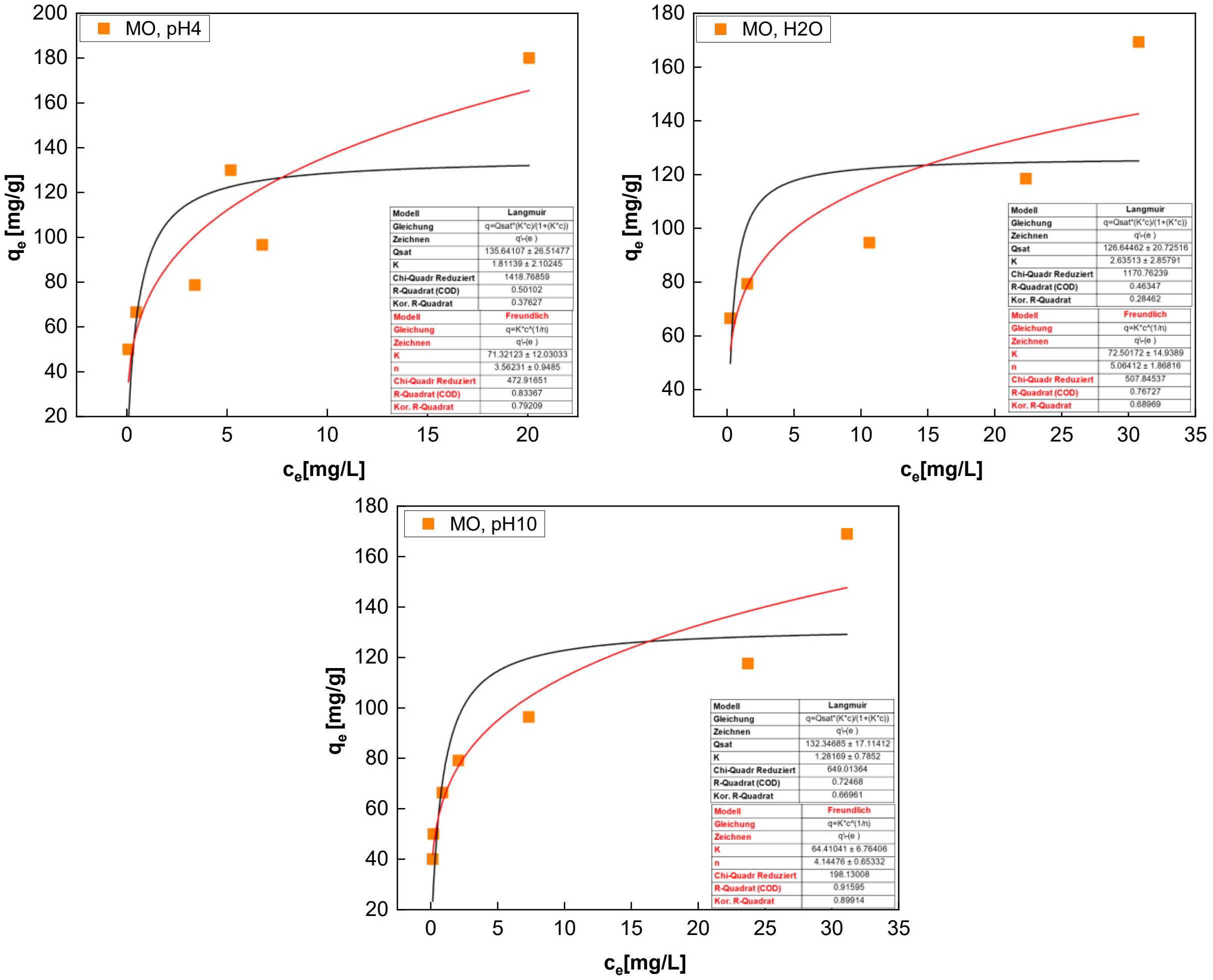

Figure S7: Adsorptions isotherms of MO at pH4 (top left), in d.i. water (top right) at pH10 (bottom) including the modelled Langmuir and Freundlich isotherms

References

(1) Block, I.; Günter, C.; Duarte Rodrigues, A.; Paasch, S.; Hesemann, P.; Taubert, A. Carbon Adsorbents from Spent Coffee for Removal of Methylene Blue and Methyl Orange from Water. *Materials (Basel, Switzerland)* **2021**, *14* (14). DOI: 10.3390/ma14143996. Published Online: Jul. 16, 2021.

(2) Beamson, G.; Briggs, D. High Resolution XPS of Organic Polymers: The Scienta ESCA300 Database (Beamson, G.; Briggs, D.). *J. Chem. Educ.* **1993**, *70* (1), A25. DOI: 10.1021/ed070pA25.5.

(3) Díez, N.; Śliwak, A.; Gryglewicz, S.; Grzyb, B.; Gryglewicz, G. Enhanced reduction of graphene oxide by high-pressure hydrothermal treatment. *RSC Adv.* **2015**, *5* (100), 81831–81837. DOI: 10.1039/C5RA14461B.

(4) Sleigh, C.; Pijpers, A. P.; Jaspers, A.; Coussens, B.; Meier, R. J. On the determination of atomic charge via ESCA including application to organometallics. *Journal of Electron Spectroscopy and Related Phenomena* **1996**, *77* (1), 41–57. DOI: 10.1016/0368-2048(95)02392-5.

(5) Dilks, A. The identification of peroxy-features at polymer surfaces by ESCA. *J. Polym. Sci. Polym. Chem. Ed.* **1981**, *19* (6), 1319–1327. DOI: 10.1002/pol.1981.170190604.
